# The Loci of Stroop Interference and Facilitation Effects With Manual and Vocal Responses

**DOI:** 10.3389/fpsyg.2019.01786

**Published:** 2019-08-19

**Authors:** Maria Augustinova, Benjamin A. Parris, Ludovic Ferrand

**Affiliations:** ^1^UNIROUEN, CRFDP, Normandie Université, Rouen, France; ^2^CNRS, LAPSCO, Université Clermont Auvergne, Clermont-Ferrand, France; ^3^Department of Psychology, Bournemouth University, Bournemouth, United Kingdom

**Keywords:** stroop interference and facilitation, response modality, task conflict, semantic conflict, response conflict

## Abstract

Several accounts of the Stroop task assume that the Stroop interference effect has several distinct loci (as opposed to a single response locus). The present study was designed to explore whether this is the case with both manual and vocal responses. To this end, we used an extended form of the Stroop paradigm (Augustinova et al., 2018b) that successfully distinguishes between the contribution of the task vs. semantic vs. response conflict to overall Stroop interference. In line with past findings, the results of Experiment 1 yielded an important response modality effect: the magnitude of Stroop interference was substantially larger when vocal responses were used (as opposed to key presses). Moreover, the present findings show that the response modality effect is specifically due to the fact that Stroop interference observed with vocal responses results from the significant contribution of task, semantic, and response conflicts, whereas only semantic and response conflicts clearly significantly contribute to Stroop interference observed with manual responses (no significant task conflict was observed). This exact pattern was replicated in Experiment 2. Also, and importantly, Experiment 2 also investigated whether and how the response modality effect affects Stroop facilitation. The results showed that the magnitude of Stroop facilitation was also larger when vocal as opposed to manual responses were used. This was due to the fact that semantic and response facilitation contributed to the overall Stroop facilitation observed with vocal responses, but surprisingly, only semantic facilitation contributed with manual responses (no response facilitation was observed). We discuss these results in terms of quantitative rather than qualitative differences in processing between vocal and manual Stroop tasks, within the framework of an integrative multistage account of Stroop interference (Augustinova et al., 2018b).

## Introduction

The typical results in the well-known Stroop task (Stroop, 1935) are at least twofold. First, Stroop *interference* refers to longer color identification times for *color-incongruent* Stroop words (i.e., words that are displayed in a color that is different from the one they designate such as “BLUE” displayed in green; hereafter *BLUE*_green_), than for *color-neutral* words (e.g., the word “DOG” displayed in green ink, hereafter *DOG*_green_) or letter strings (e.g., “XXXX” displayed in green ink, hereafter *XXXX*_green_). Second, Stroop *facilitation* refers to shorter color identification times for *color-congruent* Stroop words (i.e., *GREEN*_green_) than for *color-neutral* words (e.g., the word “DEAL” displayed in green ink, hereafter *DEAL*_green_) or letter strings (e.g., “XXXX” displayed in green ink, hereafter *XXXX*_green_).

A still unexplained finding in the Stroop literature is that the magnitude of both Stroop interference and facilitation depends on the type of response output that the Stroop task involves (MacLeod, 1991). Specifically, this magnitude is usually substantially larger when the individuals are required to identify the font color of written characters vocally (saying the color name aloud) as compared to manually (key press responses; e.g., White, 1969; Neill, 1977; Redding and Gerjets, 1977; McClain, 1983; Sharma and McKenna, 1998). Moreover, some have argued that manual and vocal responses have differential access to the systems producing interference and facilitation (Glaser and Glaser, 1989; Sugg and McDonald, 1994; Sharma and McKenna, 1998). This suggests that the way participants identify the color of Stroop stimuli determines how the different features of these compound stimuli are actually processed. This puzzling idea might explain the recently renewed interest in just this issue (Kinoshita et al., 2017; Fennell and Ratcliff, 2019; Zahedi et al., 2019; Parris et al., in press see also Parris et al., under review).

It has been argued that the manual response Stroop task is a different task to the vocal response Stroop task (Kinoshita et al., 2017). Specifically, since manual responding involves color classification and vocal responding requires color naming, the tasks differ and so then should the mechanisms that lead to Stroop interference. Such an account predicts that the locus of Stroop effects varies by response mode and finds support in influential models of the Stroop task (Glaser and Glaser, 1989; Sugg and McDonald, 1994; Sharma and McKenna, 1998). In contrast, the traditional response competition view of the Stroop task (Morton and Chambers, 1973; Cohen et al., 1990; Roelofs, 2003) has assumed that the reading task that produces Stroop effects is invariant and, thus, that the locus of the Stroop effect should be similar for manual and vocal responding.

It is clear that there are differences between the two response modes. With a manual response, the irrelevant word provides evidence toward another key press option. With the vocal response, the irrelevant word provides evidence toward another speech production option. Therefore, the ensuing Stroop interference will depend on how difficult it is to favor the correct, or inhibit the alternative, option. That the interference magnitudes with the two response modes are not equivalent suggests that suppressing the irrelevant speech code is harder than suppressing the irrelevant key press option. This is perhaps due to there being separate effectors (different fingers) for each response option with a manual response vs. a single effector (one mouth) with the vocal response. With the manual response, it is possible that a speech code is also produced for the irrelevant word, but this speech code would not interfere because there is no competing speech code associated with the relevant, correct response. It is possible then, that for both response modes, the locus is at the later stage of response selection but that response selection happens in different modules due to there being different effectors.

Alternatively, it is possible that the response mode necessarily modifies how the irrelevant word is processed and, therefore, modifies the locus of Stroop interference. It has been argued that responding vocally encourages the phonological encoding of the irrelevant word, more than the manual response (Van Voorhis and Dark, 1995; Burt, 1999; if it happens at all with a manual response – see Kinoshita et al., 2017, and Parris et al., in press), which would account for the large Stroop effects with vocal responses and supports the notion that the task itself modifies how the word is processed. However, some models of the Stroop task predict no Stroop effects at all with manual responses (Glaser and Glaser, 1989), some predict no effect with manual responses depending on the button label-type (Sugg and McDonald, 1994), and some predict differential access to semantics with manual responses (Sharma and McKenna, 1998; although see Brown and Besner, 2001). Despite these competing accounts, until recently, empirical work that addressed the issue of processes underlying this response modality effect was scarce. Also, and importantly, the recent work that has been carried out has not directly investigated established sources of conflict and, furthermore, has considered Stroop facilitation effects.

To illustrate, the recent application of the RTCON2 multichoice decision-making and confidence model (Ratcliff and Starns, 2013) to the data from the four-color Stroop tasks firmly pointed to the fact that the differences between vocal and manual response modality lie for an important part outside of the processes of decision-making (Fennell and Ratcliff, 2019, Experiment 3; see also converging evidence from the two-color choice Stroop task). However, since the RTCON2 model does not describe sources of conflict or specify processes that contribute to performance at other stages of processing in the Stroop task (i.e., all these processes are confounded in the non-decision time parameter of RTCON2), processes driving the substantial response modality effect – observed in this experiment – remain to be elucidated. Therefore, the two experiments reported in this paper were designed to shed additional light on whether manual and vocal Stroop tasks result in interference effects at different levels of processing. Specifically, we set out to investigate whether the manual and vocal response Stroop tasks produce task, semantic, and response conflict and the much-understudied effects of response and semantic facilitation.

### Varieties of Conflict and Facilitation in the Stroop Task

Several accounts of the Stroop task posit that Stroop interference results from the simultaneous contribution of two distinct conflicts. In addition to *response conflict* as depicted above, they posit the existence of the so-called *task conflict* (hereafter TC-RC accounts; see Augustinova et al., 2018b; Parris et al., under review, for reviews) instead of the semantic conflict assumed by the aforementioned SC-RC accounts. Task conflict is thought to arise for all kinds of readable items (including color-congruent words, e.g., *BLUE*_blue_) and is, thus, different from the specific color-incongruency conflict occurring for color-incongruent Stroop words (e.g., *BLUE*_green_). This is because the individual’s attention is drawn to an irrelevant task (i.e., word reading) instead of being fully focused on the relevant task (i.e., color naming), leading to the two task sets to compete (e.g., Monsell et al., 2001; Goldfarb and Henik, 2006, 2007; Kalanthroff et al., 2013a, b; Parris, 2014 for empirical demonstrations; see also, e.g., Aarts et al., 2009; Desmet et al., 2011; Elchlepp et al., 2013 for fMRI and EEG evidence).

Other accounts argue for the existence of *stimulus* (*or semantic*) *conflict*, which is thought to occur earlier in processing than the response conflict (but likely after task conflict – see Hershman and Henik, 2019). For instance, Seymour (1977) considers that this (early) conflict occurs at conceptual encoding of color-incongruent words (e.g., *BLUE*_green_) because the meaning of the word dimension (i.e., blue for *BLUE*_green_) and that of the color dimension (i.e., green here) both correspond to colors. Indeed, “(…) delays of processing occur whenever distinct semantic codes are simultaneously activated, and that these delays become acute when the conflicting codes are values on a single dimension or closely related dimensions” (p. 263; see also, e.g., Scheibe et al., 1967; Seymour, 1974; Seymour, 1977; Stirling, 1979; Luo, 1999; but see, e.g., Hock and Egeth, 1970 for the idea of perceptual rather than conceptual type of stimulus conflict). There is substantial evidence for the presence of conflict at this level of processing (Zhang and Kornblum, 1998; De Houwer, 2003; Manwell et al., 2004; Schmidt and Cheesman, 2005; Augustinova and Ferrand, 2014a; see also, e.g., van Veen and Carter, 2005; Szucs and Soltész, 2010; Chen et al., 2013; Killikelly and Szücs, 2013; Augustinova et al., 2015; for electrophysiological and fMRI evidence), although it has been proposed that stimulus conflict is an indirect measure of response conflict (Roelofs, 2003; see Parris et al., under review, for a review and evaluation of this evidence). It is, thus, not surprising that conceptualizations of multistage processing in the Stroop task assume that color-incongruent words (e.g., *BLUE*_green_) generate both stimulus and response conflicts (hereafter SC-RC accounts; see Augustinova et al., 2018b for this terminology and review of these accounts).

Given that considerable behavioral, electroencephalography (EEG), and functional magnetic resonance imaging (fMRI) evidence points to the viability of both SC-RC and TC-RC multistage accounts of Stroop interference (see above), several lines of research highlighted the necessity to adopt an integrative perspective that would allow for bridging the two previously outlined multistage perspectives (Augustinova et al., 2018b; Parris et al., under review; for reviews). To implement this latter integrative proposal empirically, Augustinova et al. (2018b); see also Ferrand et al., in press) proposed that color-associated incongruent words (e.g., *SKY*_green_) and color-neutral letter strings (e.g., *XXX*_green_) supplement the standard color-incongruent words (e.g., *BLUE*_green_) and color-neutral words (e.g., *DOG*_green_) that are commonly used in the standard Stroop task (see above). Indeed, if the color-neutral letter strings (e.g., *XXX*_green_) and words (e.g., *DOG*_green_) only trigger task conflict, the color incongruency involved in both color-associated (e.g., *SKY*_green_) and standard (e.g., *BLUE*_green_) color-incongruent words triggers additional type(s) of conflict. More specifically, color-associated incongruent words (e.g., *SKY*_green_) trigger both task and semantic conflicts, and standard color-incongruent words (e.g., *BLUE*_green_) trigger all three types of conflict (i.e., task, semantic, and response; see the section “Present Study,” for further developments).

Using this *extended form of the Stroop paradigm* – that builds on both SKY-PUT design suggested by Neely and Kahan’s (2001) and Klein’s (1964) semantic gradient – all three conflicts (i.e., task, semantic, and response conflicts) have been shown to contribute significantly to standard Stroop interference in both adults (Augustinova et al., 2018b) and reading-level children (Ferrand et al., in press) and have been shown to have specific developmental trajectories (Ferrand et al., in press). Taken together, these studies not only strongly reaffirm that the standard (i.e., overall) Stroop interference constitutes a composite and not a unitary (response-level) phenomenon but also clearly show the relevance of an integrative perspective bridging SC-RC and TC-RC multistage accounts. Yet, the extent to which these same components actually contribute to the overall Stroop interference collected with manual responses is a still-open issue. Therefore, the present study examined whether and the extent to which task, semantic, and response conflicts are affected by the type of response output (verbal vs. manual) that the Stroop task requires.

Additionally, the present study also examined how different forms of facilitation are modified by response modality. Indeed, to the best of our knowledge, only one published study has explored the potential variety in Stroop facilitation effects. Using a vocal response, Dalrymple-Alford (1972) reported a 42-ms semantic-associative facilitation effect (e.g., *DOG*_blue_ *– SKY*_blue_) and a 63-ms standard facilitation effect (e.g., *DOG*_blue_ *– BLUE*_blue_), suggesting a response facilitation effect of 21 ms. Interestingly, however, when compared to a letter string baseline (e.g., *XXX*_blue_), the congruent semantic associates actually produced interference, a finding implicating an influence of task conflict. These isolable forms of facilitation are interesting, require further study with more modern methods, and have the potential to shed light on impairments in selective attention and cognitive control. Of further interest of the present study is how these two forms of facilitation are modified by response modality.

### The Response Modality Effect Examined Within Multistage Accounts of Stroop Interference

In the aforementioned study of Augustinova et al. (2018b), the response modality effect was not an issue under consideration. Yet, the specific contributions of the *task* (e.g., *DOG*_green_ – *XXX*_green_), *semantic* (e.g., *SKY*_green_ – *DOG*_green_), and *response* conflict (e.g., *BLUE*_green_ – *SKY*_green_) to the overall Stroop interference were examined with both manual (Experiment 1) and vocal responses (Experiment 2). While the contribution of both response and semantic conflicts was significant in both experiments, with only the former being larger with the vocal response, the one of task conflict failed to reach significance when the Stroop task was administered with manual as opposed to vocal responses. Likewise, Kinoshita et al. (2017) observed task conflict with the vocal (Experiment 1) but not the manual (Experiment 2) Stroop task, but did not include a semantic Stroop condition to distinguish response and semantic conflict. However, more recently, Kinoshita et al. (2018) reported that both task and semantic conflicts were significant with both verbal (Experiments 1 and 3) and manual responses (Experiments 2 and 4), albeit with the magnitude of task conflict (but not of semantic conflict) being larger when a vocal (as opposed to manual) response output was required^1^. They did not, however, include a measure of response conflict. Thus, only one of the above studies included all three conflict types in the same study (Augustinova et al., 2018b), but none investigated facilitation types, and in all the above studies, response modality was a between-subjects factor.

There is only one study as far as we are aware that has used a within-subject design to investigate all three conflict types in both manual and vocal responses. Sharma and McKenna (1998) reported that task conflict (which they referred to as the lexical component of the Stroop effect) and semantic conflict were present when a verbal but not manual response output was required but that response conflict was present with both response types (see also, e.g., Redding and Gerjets, 1977; McClain, 1983). Sharma and McKenna’s original conclusion about the lack of semantic Stroop effects with the manual response Stroop task was based on comparisons of adjacent conditions (in terms of response times), but Brown and Besner (2001) reanalyzed Sharma and McKenna’s data using non-adjacent conditions and revealed semantic Stroop effects with manual responses. However, given that the adjacent conditions did not reveal evidence of semantic conflict, its magnitude must have differed between response modes.

In summary, of the four studies reviewed here, three provide evidence for a lack of task conflict with a manual response, but one provided evidence for the presence of task conflict with a manual response. Of the three studies designed to assess semantic conflict, all three provide evidence for semantic conflict with a manual response, but one showed greater semantic conflict with the vocal response Stroop task. Of the two studies designed to assess the individual contribution of response conflict, both provide evidence for larger response conflict with a vocal response. However, in only two of these studies were all three conflict types manipulated in the same experiment, and in only one of these studies was response modality manipulated within subjects. Notably, none of the above studies considered varieties of Stroop facilitation.

### Present Study

The present study was designed to further explore the types of conflict and facilitation and, thus, the locus of Stroop effects, with manual and vocal responses. To this end, the aim of Experiment 1 was to generalize the findings of Augustinova et al. (2018b); without the response stimulus interval manipulation, with manual and vocal responses. The aim of Experiment 2 was to extend these findings by including measures of response and semantic facilitation and by employing a fully within-subjects design.

To this end, the present study used the aforementioned *extended form of the Stroop paradigm* (Augustinova et al., 2018b). The irrelevant dimension of all stimuli included in this paradigm (i.e., color-neutral letter strings, color-neutral words, color-associated and standard color-incongruent words) is composed of letters and, thus, is assumed to generate task conflict. Importantly, they do so to the same extent, except for the non-readable color-neutral letter strings (e.g., *XXX*_green_). In line with the bimodal, interactive activation model with (amodal) semantics (McClelland and Rumelhart, 1981; McClelland, 1987; Grainger and Ferrand, 1996; Stolz and Besner, 1996; Ferrand and New, 2003; McNamara, 2005), the processing of the written dimension of these color-neutral letter strings (i.e., xxx) stops at the orthographic prelexical level. The processing of the written dimension for all other stimuli composed of words (e.g., dog, sky, and blue) stops, on the other hand, with access to meaning (i.e., after a full chain of visual, orthographic, lexical, and semantic processing has come to completion). Consequently, the significant difference in mean response latencies between Stroop color-neutral words and letter strings (e.g., *DOG*_green_ - *XXX*_green_) is thought to solely reflect differences in activation of the irrelevant reading task set and, hence, of the differential amount of the task conflict that this entails. Indeed, because the meaning of color-neutral words (e.g., dog for *DOG*_green_) is not related to a color (unlike sky or blue), the aforementioned contribution of task conflict to overall Stroop interference is not intermixed with that of the semantic and response conflicts that are generated by color incongruency.

Turning now to the separation of semantic and response conflicts and facilitation, numerous studies have argued that color incongruency causes semantic conflict (see Seymour’s reasoning outlined above). Also, and importantly, in line with Seymour (1977), semantic conflict is generated to the same extent by associated (e.g., *SKY*_green_) as compared to standard (e.g., *BLUE*_green_) Stroop words (e.g., see Augustinova et al., 2015 for N400-like evidence). Consequently, the significant difference in mean response latencies between color-associated and color-neutral trials (e.g., *SKY*_green_ – *DOG*_green_) is likely to reflect the semantic conflict that color-associated (e.g., *SKY*_green_) unlike color-neutral (*DOG*_green_) Stroop words generate. Indeed, given that color-associated words do not activate (pre-)motor responses linked to the associated color (e.g., press a blue button on seeing SKY; see Schmidt and Cheesman, 2005 for a direct demonstration), the aforementioned contribution of semantic conflict to overall Stroop interference is not confounded with that of response conflict – generated by standard color-incongruent words only (e.g., *BLUE*_green_, but see Hasshim and Parris, 2014, 2015 for a discussion of this study). Likewise, semantic facilitation with color-associated congruent stimuli (e.g., *SKY*_blue_) would not be confounded with response facilitation observed on standard congruent trials (e.g., *BLUE*_blue_).

Finally, the irrelevant word dimension of standard incongruent trials also primes the aforementioned (pre-)response tendency that – for these words (e.g., blue for *BLUE*_green_) – is part of the response set. It therefore interferes with the (pre-)response tendency primed by the meaning of the relevant color dimension (green here). Consequently, the significant difference in mean response latencies between standard and associated color-incongruent trials (e.g., *BLUE*_green_ – *SKY*_green_) is thought to result from this (pre-)motor (i.e., response) conflict occurring at the level of response processing and/or output. Likewise, the difference between color-associated congruent trials and standard congruent trials (e.g., *SKY*_blue_ – *BLUE*_blue_) would represent response facilitation. Indeed, both task and semantic conflicts are assumed to be equal in those two types of color-incongruent items (*BLUE*_green_ and *SKY*_green_, see above) even though more complex interactions between these different conflicts cannot be excluded.

To sum up, in both Experiments 1 and 2, the positive difference in mean response latencies between standard color-incongruent words and color-neutral letter strings (e.g., *BLUE*_green_ – *XXX*_green_) was used to measure the magnitude of overall Stroop interference. Furthermore, in both experiments (and as in Augustinova et al., 2018b’s study), the positive difference in mean response latencies between color-neutral words and letter strings (e.g., *DOG*_green_ – *XXX*_green_) was used as a proxy for assessing the specific contribution of *task* conflict to this overall Stroop interference. The positive difference in mean response latencies between color-associated incongruent and color-neutral trials (e.g., *SKY*_green_ – *DOG*_green_) was used as a proxy for assessing the specific contribution of *semantic* conflict to overall Stroop interference. Finally, the positive difference in mean response latencies between standard color-incongruent and color-associated incongruent trials (e.g., *BLUE*_green_ – *SKY*_green_) was used as a proxy for assessing the specific contribution of *response* conflict to overall Stroop interference.

In Experiment 2, the magnitude of overall Stroop *facilitation* was also measured. It corresponded to the positive difference in mean response latencies between color-neutral words and standard color-congruent words (e.g., *DOG*_blue_ *– BLUE*_blue_). Furthermore, the positive difference in mean response latencies between color-neutral trials and color-associated congruent trials (e.g., *DOG*_blue_ – *SKY*_blue_) was used to isolate the specific contribution of *semantic* facilitation to the aforementioned overall Stroop facilitation. Finally, the positive difference in mean response latencies between color-associated and standard color-congruent trials (e.g., *SKY*_blue_ – *BLUE*_blue_) was used to capture the specific contribution of *response* facilitation to overall Stroop facilitation.

The implementations of the Stroop paradigm depicted above, thus, enabled us to further assess the nature of processes that are influenced by the variations in the response output commonly employed in the Stroop task. To this end, color identification items were collected with both vocal and manual responses in both experiments. The response modality varied between participants in Experiment 1 and within participants in Experiment 2. Given the important discrepancies between findings regarding *whether* and *the extent to which* task and semantic conflict occur, respectively, with manual and vocal responses, we only *a priori* predicted that in both studies, the magnitude of Stroop interference will be larger with vocal as compared to manual responses and that this difference should result at least in part from a difference in response conflict.

## Experiment 1

### Method

#### Participants

Seventy-six psychology undergraduates (56 females and 10 males, all native French speakers reporting normal or corrected-to-normal vision, M_ag__e_ = 19.5 years; M_mi__n_ = 18; M_mi__n_ = 24) at Université Clermont Auvergne, Clermont-Ferrand, France, took part in this experiment in exchange for a course credit. The data of four participants were excluded from the analyses^2^, leaving a total of 72 participants (38 in the manual and 34 vocal response modality).

#### Design and Stimuli

Since the participants were randomly assigned to one of the two response modality conditions, the data were collected using a 2 (response modality: manual vs. vocal) × 4 (stimulus type: color-incongruent words vs. color-associated words vs. color-neutral words vs. color-neutral signs) design, with the first of these being used as a between-participants factor. There were 60 trials for each stimulus-type factor condition (resulting from five repetitions of the same set of stimuli), which varied randomly within a single block of 240 experimental trials.

The stimuli (presented in lowercase Courier font, size 18, on a black background) consisted of four color words: *rouge* [red], *jaune* [yellow], *bleu* [blue], and *vert* [green]; four color-associated words: *tomate* [tomato], *maïs* [corn], *ciel* [sky], and *salade* [salad]; four color-neutral words: *balcon* [balcony], *robe* [dress], *pont* [bridge], and *chien* [dog]; and strings of Xs of the same length as the color-incongruent trials. The four color-associated words were selected as strong associates (tomato-red: 49.4%; corn-yellow: 30.2%; sky-blue: 44%; and salad-green: 31.5%) from French word association norms (Ferrand and Alario, 1998; De La Haye, 2003) and pretested as depicted in Augustinova and Ferrand (2007). In each condition, all the stimuli were similar in length (4.5, 5, 4.75, and 4.75 letters on average for the color-incongruent words, the color-associated words, the color-neutral words, and the strings of Xs, respectively) and frequency (74, 82, and 84 occurrences per million for the color-incongruent words, the color-associated words, and the color-neutral words, respectively) according to Lexique (New et al., 2004). Color-incongruent and color-associated items always appeared in colors that were incongruent with the meaning of their word dimension.

#### Apparatus and Procedure

EPrime 2.1 (Psychology Software Tools, Pittsburgh, PA, United States) running on a PC (Dell Precision) was used for stimulus presentation and data collection. The participants who were tested individually were seated approximately 50 cm from a 17-inch Dell color monitor. With both response modalities, their task was to identify the color of letter strings presented on the screen as quickly and accurately as possible while ignoring their meanings. To this end, they were instructed to concentrate on the white fixation cross (“ + ”) that appeared in the center of the (black) screen at the beginning of each trial. After 500 ms, the fixation point was replaced by the stimulus that continued to be displayed until the participant responded or until 2,000 ms had elapsed.

In the manual response modality, the participants were required to respond on a keyboard placed on a table between them and the monitor. The response keys were labeled with colored stickers such that a red, blue, yellow, and green round-shaped sticker covered, respectively, the “S,” “D,” “K,” and “L” keys of an AZERTY-type keyboard. Consequently, the participants pressed the “red” key with the middle finger and “blue” key with the index finger of their left hand, and the “yellow” key with the index finger and “green” key with the middle finger of their right hand. In the vocal response modality, the participants were required to respond out loud. Their responses were recorded via a Koss 70-dB microphone headset and stored on a Sony IC Recorder-ICD PX333.

Before the beginning of the experimental block, the participants were familiarized with specificities of a given response modality. Following MacLeod (2005), 128 key-matching practice trials were used in the manual response modality so the participants can adequately learn the key–color correspondence. In the vocal response modality, the number of practice trials was reduced to 32 items. In both conditions, these practice trials consisted of strings of asterisks (presented in four aforementioned colors).

### Results and Discussion

Latencies greater than 3 SDs above or below each participant’s mean latency for each condition (i.e., less than 2% of the total data in the task administered with manual responses and less than 3% of the total data in the task-administered with oral responses) were excluded from the analyses.

Mean reaction times for correctly identified items were subsequently analyzed in the 4 (stimulus type: standard color-incongruent words vs. associated color-incongruent words vs. color-neutral words vs. color-neutral signs) × 2 (response modality: manual vs. vocal) analysis of variance (ANOVA) (see Table 1 for descriptive statistics). This analysis revealed the significant main effect of stimulus type [*F*(3,210) = 142.40; *p* < 0.001, $\eta_{p}^{2}$ = 0.670] and a marginally significant one of response modality [*F*(1,70) = 2.88; *p* = 0.094, $\eta_{p}^{2}$ = 0.039]. This latter effect was due to the fact that color identification times tended to be faster for vocal compared to manual responses. The latter main effects were also included in the significant stimulus type × response modality interaction [*F*(3,210) = 15.33; *p* < 0.001, $\eta_{p}^{2}$ = 0.180]^3^.

**TABLE 1:** Mean correct response times (in milliseconds), standard errors (in parentheses), and percentages of errors observed as a function of stimulus type and response modality.

		**Experiment 1 (between SS)**		**Experiment 2 (within SS)**	
			
**Stimulus type**		**M (*N* = 38)**	**V (*N* = 34)**	**M (*N* = 36)**	**V (*N* = 36)**
Standard Color-incongruent words *BLUE*_green_	RT %Errors	815 (23) 1.75	809 (25) 4.56	733 (21) 3.06	819 (22) 7.11
Associated Color-incongruent words *SKY*_green_	RT %Errors	772 (19) 1.23	730 (20) 1.13	683 (16) 2.54	718 (16) 1.27
Color-neutral words *DOG*_green_	RT %Errors	747 (17) 1.19	701 (18) 0.78	665 (16) 1.91	695 (15) 0.69
Color-neutral signs *XXXX*_green_	RT %Errors	739 (16) 1.33	653 (17) 0.49	660 (15) 1.73	651 (13) 0.23
Associated Color-congruent words *SKY*_blue_	RT %Errors	ni	ni	651 (15) 1.96	684 (14) 0.40
Standard Color-congruent words *BLUE*_blue_	RT %Errors	ni	ni	644 (15) 1.79	645 (15) 0.28

*M, manual; V, vocal; ni, not included.*

Its decomposition further revealed that the simple main effect of stimulus type was significant in both manual [*F*(3,68) = 17.83; *p* < 0.001, $\eta_{p}^{2}$ = 0.440] and vocal [*F*(3,68) = 53.61; *p* < 0.001, $\eta_{p}^{2}$ = 0.703] response modalities. Additional contrast analyses of these simple main effects revealed that in both response modalities, latencies for standard color-incongruent words were significantly longer than those observed for color-neutral signs (both *p*_s_ < 0.001). Thus, a substantial amount of Stroop interference (i.e., *BLUE*_green_ – *XXX*_green_) occurred in both response modalities. Yet, latencies for color-neutral words were significantly longer than those observed for color-neutral signs (see Tables 1, 2) only in the vocal (*p* < 0.001) but not in the manual (*p* = 0.159; M_differenc__e_ = 7 ms; 95%CI = −3 to 18) response modality. This latter result implies that, in the Stroop task administered with manual responses, the contribution of task conflict to the overall Stroop interference failed to reach significance, whereas the contribution of both semantic (i.e., *SKY*_green_ – *XXX*_green_) and response conflicts (i.e., *BLUE*_green_ – *SKY*_green_) was significantly independently of the response output that was required (all *p*_s_ < 0.001).

**TABLE 2:** Stroop-like effects (in milliseconds and percent ratios) observed as a function of response modality.

		**Experiment 1 (between SS)**				**Experiment 2 (within SS)**			
			
**Stroop-like effects**		**M**		**V**	**Resp. Modality effect**	**M**		**V**	**Resp. modality effect**
Standard Stroop interference *BLUE*_green_ *– XXXX*_green_	RT diff.	76^∗^	<	155^∗^	**79^∗^**	73^∗^	<	168^∗^	**95^∗^**
	
	Percent ratio	0.097^∗^	<	0.218^∗^	**0.121^∗^**	0.106^∗^	<	0.239^∗^	**0.133^∗^**

Response Conflict *BLUE*_green_ *– SKY*_green_	RT diff.	44^∗^	<	78^∗^	**34^∗^**	50^∗^	<	101^∗^	**51^∗^**
	
	Percent ratio	0.055^∗^	<	0.104^∗^	**0.048^∗^**	0.071^∗^	<	0.138^∗^	**0.067^∗^**

Semantic Conflict *SKY*_green_ *– DOG*_green_	RT diff.	25^∗^	≈	29^∗^	**4^ns^**	18^∗^	≈	23^∗^	**5^ns^**
	
	Percent ratio	0.033^∗^	≈	0.039^∗^	**0.006^ns^**	0.028^∗^	≈	0.033^∗^	**0.005^ns^**

Task Conflict *DOG*_green_ *– XXX*_green_	RT diff.	7^ns^	<	48^∗^	**41^∗^**	5^ns^	<	44^∗^	**39^∗^**
	
	Percent ratio	0.009^ns^	<	0.075^∗^	**0.066^∗^**	0.007^ns^	<	0.068^∗^	**0.061^∗^**

Semantic Facilitation *DOG*_blue_ *– SKY*_blue_	RT diff.	ni		ni		14^*a*^	≈	11^*b*^	**-3^ns^**
	
	Percent ratio	ni		ni		0.022^*b*^	≈	0.016^*b*^	**-0.006^ns^**

Response Facilitation *SKY*_blue_ *– BLUE*_blue_	RT diff.	ni		ni		7^ns^	<	39^∗^	**32^∗^**
	
	Percent ratio	ni		ni		0.013^ns^	<	0.063^∗^	**0.050^∗^**

Standard Stroop facilitation *DOG*_blue_ *– BLUE*_blue_	RT diff.	ni		ni		21^∗^	<	50^∗^	**29^∗^**
	
	Percent ni ratio	ni		ni		0.035^∗^	<	0.079^∗^	**0.044^∗^**

*^∗^*p* < 0.001; ^*a*^*p* = 0.008; ^*b*^*p* < 0.01; M, manual; V, vocal; RT diff., reaction time differences; ni, not included. Bold values correspond to the Response Modality Effect.*

To examine further the extent to which the variation in response modality specifically influences task vs. semantic vs. response conflict, magnitudes of these conflicts were analyzed in 3 (conflict type: task vs. semantic vs. response) × 2 (response modality: manual vs. vocal) ANOVA (see Table 2). This analysis revealed significant main effects of conflict type [*F*(2,140) = 20.46; *p* < 0.001, $\eta_{p}^{2}$ = 0.226] and of response modality [*F*(1,70) = 21.72; *p* < 0.001, $\eta_{p}^{2}$ = 0.237] that were also included in the significant conflict type × response modality interaction [*F*(2,140) = 5.10; *p* = 0.007, $\eta_{p}^{2}$ = 0.068]. Its decomposition further revealed that the simple main effect of response modality was significant on task [*F*(1,70) = 29.54; *p* < 0.001, $\eta_{p}^{2}$ = 0.297] and response conflicts [*F*(1,70) = 8.18; *p* = 0.006, $\eta_{p}^{2}$ = 0.105], such that their contribution to the overall interference was significantly larger when vocal (as opposed to manual) response output was required (see Table 2). This latter variation in the response output failed to influence the magnitude of semantic conflict [*F*(1,70) = 0.40; *p* = 0.532, $\eta_{p}^{2}$ = 0.006; M_differenc__e_ = 4 ms; 95%CI = −17 to 9]. The contribution of the latter conflict to the overall interference was significant but remained of the same magnitude with both types of the required response output (see Table 2).

Given the important differences in the speed of processing across the two types of response output, the previous analyses were supplemented by those of distinct conflict computed in the form of interference ratio (Augustinova et al., 2018a). Such that for each individual, the observed magnitude of response conflict for instance was divided by its appropriate baseline (*BLUE*_green_ – *SKY*_green_/*SKY*_green_). Resulting ratios were subsequently analyzed in 3 (conflict-type percent: task vs. semantic vs. response) × 2 (response modality: manual vs. vocal) ANOVA (see Table 2). This analysis mirrored the aforementioned results, such that it revealed significant main effects of conflict type [*F*(2,140) = 16.49; *p* < 0.001, $\eta_{p}^{2}$ = 0.191] and of response modality [*F*(1,70) = 38.26; *p* < 0.001, $\eta_{p}^{2}$ = 0.353] that were also included in the significant conflict type × response modality interaction [*F*(2,140) = 7.07; *p* = 0.001, $\eta_{p}^{2}$ = 0.092]. Its decomposition further revealed that the simple main effect of response modality was significant on the ratio of task [*F*(1,70) = 37.97; *p* < 0.001, $\eta_{p}^{2}$ = 0.352] and of response conflict [*F*(1,70) = 11.02; *p* = 0.001, $\eta_{p}^{2}$ = 0.136], such that their contribution to the overall interference was significantly larger when vocal (as opposed to manual) response output was required (see Table 2). Again, this latter variation in the response output failed to influence the ratio of semantic conflict [*F*(1,70) = 0.56; *p* = 0.458, $\eta_{p}^{2}$ = 0.008; M_differenc__e_ = −0.006; 95%CI = −0.023 to 0.010].

In line with past literature, the results reported show substantially larger magnitudes of Stroop interference with vocal as compared to manual responses (see Table 2). These differences were due to the fact that both response and task conflict contributed less when manual response output was required – to the point that the contribution of task conflict remained non-significant in this response modality replicating Augustinova et al. (2018b); Experiment 1. The contribution of semantic conflict to overall Stroop interference remained significant, but the size of its magnitude remained equivalent across the two types of response output (see Table 2).

These results have several potentially interesting implications. First, the possible absence of task conflict in the Stroop task administered with manual responses, at least when measured by comparing response to color neutral and repeated Xs baseline, suggests that qualitative (Sharma and McKenna, 1998; Kinoshita et al., 2017) rather than just quantitative (Brown and Besner, 2001; Augustinova et al., 2018b; Parris et al., in press) differences between response modes. Thus, the investigation of the response modality effect in the Stroop task might actually add to uncovering the different components of Stroop interference observed with manual as compared to vocal responses supporting the notion that the two tasks are not equivalent (e.g., naming vs. categorization task entailing the different processes; see, e.g., Kinoshita et al., 2017; Fennell and Ratcliff, 2019). Given the importance of this second implication of the results reported, the following experiment was designed to (a) replicate these results while the response modality was manipulated within participants, and (b) extend them to Stroop facilitation (i.e., difference in mean reaction times for color-neutral and standard color-congruent words; *DOG*_green_ – *BLUE*_blue_).

The rationale behind this extension corresponds to a further investigation of the fact that the magnitude of semantic conflict remained equivalent with both response modalities (see also Augustinova et al., 2018b but see Sharma and McKenna, 1998; Brown and Besner, 2001; Kinoshita et al., 2018). If semantic processing in the Stroop task is indeed invariant (and as such it cannot be prevented and/or reduced), results on Stroop facilitation should logically mirror those observed in the present experiment on Stroop interference. More specifically, Stroop facilitation observed with both manual and vocal responses should result from a substantial amount of semantic facilitation (i.e., differences in mean reaction times for color-neutral words and associated color-congruent words, e.g., *DOG*_green_ – *SKY*_blue_) that should arise in both response modalities. However, the contribution of response facilitation (i.e., differences in mean reaction times for associated and standard color-congruent words; e.g., *SKY*_green_ – *BLUE*_blue_) should be reduced in manual response modality. The following experiment was designed to test just these predictions.

## Experiment 2

### Method

Forty-five psychology undergraduates (36 females and 9 males, all native French speakers reporting normal or corrected-to-normal vision, M_ag__e_ = 21.04 years; M_mi__n_ = 19; M_mi__n_ = 26) at Université Clermont Auvergne, Clermont-Ferrand, France, took part in this experiment in exchange for a course credit. The data of nine participants were excluded from the analyses^4^, leaving a total of 36 participants. Unlike in Experiment 1, response modality factor was manipulated within participants. Thus, the order of the two response modalities was counterbalanced in a random fashion, such as half of the participants responded with a manual, the other half with a vocal response modality first. Stimulus-type factor used in Experiment 1 was supplemented with two new kinds of items: standard color-congruent (*BLUE*_blue_) and associated color-congruent (*SKY*_blue_) words. In other words, color words not only appeared in colors that were incongruent but also congruent with the meaning of their word dimension. There were 48 trials in each condition of stimulus-type factor (resulting from four repetitions of the same set of color-incongruent and color-neutral stimuli and 12 repetitions of the same set of color-congruent stimuli) that varied randomly within a single block of 288 experimental trials (that was executed in each of the two response modalities). Because of this balancing, the facilitation effect was subject to a contingency bias (Schmidt and Besner, 2008; Schmidt et al., 2015). However, our main interest lies in processes underlying the response modality effect; thus, the same randomization procedure was used (see also, e.g., Fennell and Ratcliff, 2019).

Finally, DMDX software (Forster and Forster, 2003) was used for stimulus presentation and data collection. Remaining aspects were identical to those depicted in the section “Methods” for Experiment 1, including the practice trials that were administered again before the beginning of each of the two experimental blocks.

### Results and Discussion

Latencies greater than 3 SDs above or below each participant’s mean latency for each condition (i.e., less than 1% of the total data in the task administered with manual responses and less than 2% of the total data in the task-administered with oral responses) were excluded from the analyses.

Mean reaction times for correctly identified items were first analyzed in the omnibus 6 (stimulus type: standard color-incongruent words vs. associated color-incongruent words vs. color-neutral words vs. color-neutral signs vs. associated color-congruent words vs. standard color-congruent words) × 2 (response modality: manual vs. vocal) ANOVA (see Table 1 for descriptive statistics). This analysis revealed the significant main effects of stimulus type [*F*(5,175) = 113.65; *p* < 0.001, $\eta_{p}^{2}$ = 0.765] and of response modality [*F*(1,35) = 6.40; *p* = *0.016*, $\eta_{p}^{2}$ = 0.155]. This latter effect was due to faster color identification times for manual compared to vocal responses. The latter main effects that were also included in a stimulus × response modality [*F*(5,175) = 27.92; *p* < 0.001, $\eta_{p}^{2}$ = 0.444]^5^.

The decomposition of stimulus × response modality interaction (see above) revealed that the simple main effect of stimulus type was significant in both manual [*F*(5,31) = 18.42; *p* < 0.001, $\eta_{p}^{2}$ = 0.748] and vocal [*F*(5,31) = 44.39; *p* < 0.001, $\eta_{p}^{2}$ = 0.877] response modalities. Additional contrast analyses of these simple main effects revealed that in both response modalities, latencies for standard color-incongruent words were significantly longer than those observed for color-neutral signs (both *p*_s_ < 0.001), suggesting that a significant Stroop interference occurred with both types of response output.

As in Experiment 1, latencies for color-neutral words were significantly longer than those observed for color-neutral signs (see Table 1) in the vocal (*p* < 0.001) but not in the manual (*p* = 0.145; M_differenc__e_ = 5 ms; 95%CI = 2–12) response modality. This suggests again the absence of the significant contribution of the task conflict in this latter response modality, whereas both semantic (e.g., *SKY*_green_ – *DOG*_green_) and response conflicts (e.g., *BLUE*_green_ – *SKY*_green_) significantly contributed to Stroop interference in both response modalities (all *p*_s_ < 0.001).

Additionally, these contrast analyses revealed that latencies for both color-neutral words were significantly longer than those observed for standard color-congruent items (both *p*_s_ < 0.001), suggesting that a significant Stroop facilitation (e.g., *DOG*_green_ – *BLUE*_blue_) occurred with both types of response output. The additional contrast analyses revealed that under both response modalities, the Stroop facilitation resulted from a significant contribution of semantic facilitation (e.g., *DOG*_green_ *– SKY*_blue_; all *p*_s_ < 0.01) modality, whereas the contribution of response facilitation (e.g., *SKY*_bleu_ *– BLUE*_blue_) failed to reach when manual responses were used (*p* = 0.219; M_differenc__e_ = 8 ms; 95%CI = −20 to 5), while it was significant and of great magnitude (see Table 2) when vocal response output was required.

#### The Influence of Response Modality on Distinct Components of Stroop Interference

To examine further the extent to which the variation in response modality specifically influences task vs. semantic vs. response conflict, as in Experiment 1, magnitudes of these conflicts were analyzed in 3 (conflict type: task vs. semantic vs. response) × 2 (response modality: manual vs. vocal) ANOVA (see Table 2). This analysis revealed significant main effects of conflict type [*F*(2,70) = 35.00; *p* < 0.001, $\eta_{p}^{2}$ = 0.500] and of response modality [*F*(1,35) = 63.60; *p* < 0.001, $\eta_{p}^{2}$ = 0.645] that were also included in the significant conflict type × response modality interaction [*F*(2,70) = 8.65; *p* < 0.001, $\eta_{p}^{2}$ = 0.198]. Its decomposition further revealed that the simple main effect of response modality was significant on task [*F*(1,35) = 37.67; *p* < 0.001, $\eta_{p}^{2}$ = 0.518] and response conflicts [*F*(1,35) = 25.05; *p* < 0.001, $\eta_{p}^{2}$ = 0.417], such that their contribution to the overall interference was significantly larger when vocal (as opposed to manual) response output was required (see Table 2). This latter variation in the response output failed to influence the magnitude of semantic conflict [*F*(1,35) = 0.76; *p* = *0.388*, $\eta_{p}^{2}$ = 0.006; M_difference_ = -5 ms; 95%CI = −17 to 7]. Recall that the contribution of the latter conflict to the overall interference was significant but remained of the same magnitude with both types of the required response output (see Table 2).

Given the important differences in the speed of processing across the two types of response output even within participants, and as in Experiment 1, the previous analyses were supplemented by those of conflicts computed as interference ratios. Resulting ratios were subsequently analyzed in 3 (Conflict-type percent: task vs. semantic vs. response) × 2 (Response modality: manual vs. vocal) ANOVA (see Table 2). This analysis mirrored the aforementioned results. Such that it revealed significant main effects of Conflict-type [*F*(2,70) = 32.98; *p* < 0.001, $\eta_{p}^{2}$ = 0.485] and of Response-modality [*F*(1,35) = 72.65; *p* < 0.001, $\eta_{p}^{2}$ = 0.675] that were also included in the significant Conflict-type × Response-modality interaction [*F*(2,70) = 8.96; *p* < 0.001, $\eta_{p}^{2}$ = 0.204]. Its decomposition further revealed that the simple main effect of Response-modality was significant on the ratio of task [*F*(1,35) = 41.95; *p* < 0.001, $\eta_{p}^{2}$ = 0.545] and of response conflict [*F*(1,35) = 24.05; *p* < 0.001, $\eta_{p}^{2}$ = 0.407] such that their contribution to the overall interference was significantly larger when vocal (as opposed to manual) response output was required (see Table 2). Again, this latter variation in the response output failed to influence the ratio of semantic conflict [*F*(1,35) = 0.33; *p* = 0.569, $\eta_{p}^{2}$ = 0.009; M_differenc__e_ = −0.005; 95%CI = **−**0.022 to 0.012].

#### The Influence of Response Modality on Distinct Components of Stroop Facilitation

To examine further the extent to which the variation in response modality specifically influenced the contribution of semantic vs. response facilitation to the overall Stroop facilitation, magnitudes of these facilitation effects were analyzed in 2 (facilitation type: semantic vs. response) × 2 (response modality: manual vs. vocal) ANOVA (see Table 2). This analysis revealed significant main effects of response modality [*F*(1,35) = 10.94; *p* = 0.002, $\eta_{p}^{2}$ = 0.238] and of facilitation type [*F*(1,35) = 18.19; *p* < 0.001, $\eta_{p}^{2}$ = 0.342]. The facilitation type × response modality interaction was also significant [*F*(1,35) = 55.341; *p* < 0.001, $\eta_{p}^{2}$ = 0.613]. It was also significant when these latter effects were analyzed as facilitation ratios. More specifically, with these latter indicators, the main effect of facilitation type was non-significant [*F*(1,35) = 2.42; *p* = 0.128, $\eta_{p}^{2}$ = 0.065], whereas the one of response modality [*F*(1,35) = 18.41; *p* < 0.001, $\eta_{p}^{2}$ = 0.345] was significant. As already mentioned, facilitation type × response modality interaction [*F*(1,35) = 12.24; *p* = 0.001, $\eta_{p}^{2}$ = 0.259] was significant. The further decomposition of this latter interaction revealed that the simple main effect of response modality was significant on the ratio of response facilitation [*F*(1,35) = 22.73; *p* < 0.001, $\eta_{p}^{2}$ = 0.394], such that its contribution to overall Stroop facilitation was significantly larger when vocal (as opposed to manual) response output was required (see Table 2). This latter variation in the response output failed to influence the ratio of semantic facilitation [*F*(1,35) = 0.41; *p* = 0.529, $\eta_{p}^{2}$ = 0.011; M_differenc__e_ = 0.006; 95%CI = −0.012 to 0.022], such that the semantic facilitation contributed significantly to overall Stroop facilitation phenomenon in both response modalities (see Table 2 for the very same pattern of results observed with magnitudes of semantic vs. response facilitation).

It is important to note that the aforementioned pattern of results would have been diluted by the use of color-neutral signs (as opposed to words) as a baseline. Indeed, even though color-neutral signs and words are often used interchangeably as baselines, standard Stroop facilitation observed in vocal response modality (*p* = 0.558; M_differenc__e_ = 5 ms; 95%CI = 12–24 ms) and semantic facilitation observed in manual response modality (*p* = 0.139; M_differenc__e_ = 9 ms; 95%CI = 3–20 ms) would have no longer been significant if color-neutral signs were used to compute these contrasts (see, e.g., Redding and Gerjets, 1977; Brown, 2011 for other empirical demonstrations). Thus, these results are compatible with Brown’s (2011) conclusion that if a baseline consists of color-neutral signs instead of words, not only is the magnitude of Stroop interference overestimated, but also, and importantly, the magnitude of Stroop facilitation is largely underestimated. In light of the present results, but also because some task conflict actually occurs even for color-congruent stimuli (Goldfarb and Henik, 2007), it still seems useful to nuance this latter conclusion by specifying that if a baseline consists, indeed, of color-neutral signs instead of words, the magnitude of the color incongruency effect is overestimated and, importantly, the magnitude of Stroop facilitation is largely underestimated.

## General Discussion

The results of Experiments 1 and 2 yielded an important response modality effect – the direction of which was consistent with past findings (e.g., White, 1969; Redding and Gerjets, 1977; Neill, 1977; McClain, 1983; Sharma and McKenna, 1998; Kinoshita et al., 2018; Fennell and Ratcliff, 2019; Zahedi et al., 2019; Parris et al., in press). Indeed, in both experiments reported above, the magnitude of Stroop interference was substantially larger when vocal responses as opposed to key presses were used. This means that both the Stroop interference effects and response modality effects are the same in both experiments and are, therefore, not affected by the inclusion of the additional congruent conditions in Experiment 2.

The present study further extended the past results in several important ways. Indeed, it has shed a more direct light on processes driving this effect. Specifically, results of both experiments showed that the response modality effect is due to a significantly lesser contribution of task and response conflicts (but not the one of semantic conflict) to overall Stroop interference when manual, as opposed to vocal response, output is required. Even more precisely, with key presses, the magnitude of task conflict is reduced to the point that it actually fails to contribute significantly to overall Stroop interference. The significantly reduced magnitude of response conflict contributed, on the other hand, to overall Stroop interference, exactly like the magnitude of semantic conflict that remained unchanged by the induced differences in the required response output. The aforementioned pattern of results occurred independently of whether the response modality was manipulated between (Experiment 1) or within (Experiment 2) participants.

These results therefore present several potentially important implications. First, they seem consistent with a rather puzzling idea – mentioned earlier (see, section “Introduction”) – that the way participants identify the color of Stroop stimuli determines *how* (rather than *the extent to which*) different features of these compound stimuli are actually processed. Indeed, if all types of conflict (task, semantic, and response conflicts) seem to significantly contribute to the overall Stroop interference observed with vocal responses, only semantic and response conflicts clearly significantly contribute to Stroop interference observed with manual responses. Consequently, the second important implication of this pattern of results is that vocal and manual Stroop tasks might actually correspond to two different tasks. Specifically, in line with conclusions of several recent studies, the former might correspond to a naming task, whereas the latter to a categorization task, hence entailing qualitative rather than quantitative differences in processing (e.g., Kinoshita et al., 2017; Fennell and Ratcliff, 2019).

While this latter possibility remains plausible and therefore should be thoroughly addressed by additional studies, we are inclined to argue in favor of a, perhaps, more parsimonious possibility of quantitative, rather than qualitative, differences in processing between vocal and manual Stroop tasks (Roelofs, 2003). Indeed, in line with an integrative perspective that bridges both SC-RC and TC-RC multistage accounts of Stroop interference (Augustinova et al., 2018b; Parris et al., under review; for reviews), it still remains equally plausible that some amount of task conflict occurs with both types of response output. However, given a modest magnitude of this contribution with manual responses, response time might not be the most suitable indicator for capturing it (see, e.g., Augustinova et al., 2015; see also Kinoshita et al., 2018 for findings consistent with this latter conclusion). This latter reasoning is consistent with findings of Heil et al. (2004) in a letter search priming paradigm. They convincingly demonstrated that the absence of semantic activation cannot be validly inferred from the lack of response time effects in this latter paradigm. Indeed, in their study, the absence of response time effect occurred while event-related potential (ERP) correlate of semantic activation (i.e., the N400 amplitude) was still significant and sensitive to experimental manipulations used. It therefore remains possible that the significant contribution of an early component of Stroop interference such as task conflict can still be found in electrophysiological measures such as ERPs (see Elchlepp et al., 2013 for ERP task set conflict correlates observed in a version of task-switching paradigm).

Another point to note is that the measure of task conflict used in this and the previous studies mentioned above (e.g., *DOG*_green_ - *XXX*_green_) is not the only measure of task conflict. To investigate the potential role of conflict between task sets in the Stroop task, Goldfarb and Henik (2007); see also Kalanthroff et al. (2013a,b) reported a study in which they attempted to reduce task conflict control by increasing the proportion of non-word neutral trials (repeated letter strings) to 75%. Increasing the proportion of non-word neutral trials would create the expectation for a low task conflict context, and so, task conflict monitoring would effectively be offline. In addition to increasing the proportion of non-word neutral trials, on half of the trials, participants received cues that indicated whether the following stimulus would be a non-word or a color word, giving another indication as to whether the mechanisms that control task conflict should be activated. For non-cued trials, when presumably task conflict control was at its nadir, and therefore task conflict at its peak, RTs were slower for congruent trials than for non-word neutral trials, producing a negative facilitation effect. This measure of negative facilitation, indicating the presence of task conflict, was observed with a manual response. Thus, our argument is not that there is no task conflict with manual responses, but that our data provide evidence for larger task conflict with a vocal response, which would contribute to the difference in interference (and facilitation) effects often reported between the two response modes. The fact that observing negative facilitation requires an experimental manipulation that would modify facilitation and other forms of conflict (e.g., Kinoshita et al., 2018) means that it is not ideal when measuring the contribution of conflict and facilitation types to Stroop effects.

The present behavioral findings also suggest that the contribution of semantic conflict remains unaffected by variations in response modality. Several past ERP studies are in line with this result as they show that the amplitude of the aforementioned N400 – corresponding to an ERP correlate of semantic conflict (Augustinova et al., 2015) – also remains unaffected by the response modality (Liotti et al., 2000; Zahedi et al., 2019). Note, however, that the scalp distribution of N400 might eventually differ as a function of response output (Liotti et al., 2000). Taken together, the present and past results are consistent with the idea that semantic processing in the Stroop task occurs and to the same magnitude irrespective of the type of response output required (Augustinova and Ferrand, 2014a, b; Brown and Besner, 2001 for discussions, but see Sharma and McKenna, 1998; Hasshim and Parris, 2014, 2015 for different empirical findings). This latter idea is actually strengthened by the fact that semantic facilitation remained unaffected by the type of response output, whereas response facilitation was substantially reduced (to the point of its actual elimination) in manual response modality. This finding is inconsistent with the notion that Stroop effects observed with semantic Stroop stimuli are due to the indirect measurement of response conflict (Roelofs, 2003). That is, it has been argued that the connections that semantically related stimuli have to response colors (i.e., *sky* is related to blue, and it is the activation of the response blue that leads to the Stroop effect) is what leads to apparent semantic Stroop effects. In the present data, semantic Stroop effects are unaffected by response mode, whereas response conflict is affected. If semantic effects were due to connections at the response level, one would expect to see simultaneous modification of the semantic- and response-level effects. However, the semantic Stroop effects are much smaller than the response effects, and so, the preserved effects could be due to effect of magnitude as opposed to effect type (see Parris et al., under review, for a fuller discussion of this issue).

It is clear from the present data set that, as a percentage of overall Stroop interference and facilitation effects, response processing contributes less when using a manual response (compared to a vocal response), suggesting that the makeup of Stroop effects differs between response modes. This is, however, due to the substantially reduced amount of both response conflict and response facilitation. Indeed, the finding of eliminated response facilitation with the manual response is important and surprising and one that shows how facilitation with manual and vocal responses is quite different. Response facilitation then, like response conflict, is substantially reduced with manual responses, suggesting a commonality between Stroop interference and facilitation, indices that have recently been considered as potentially unrelated phenomena (Parris, 2014; see Brown, 2011 for a further discussion of this important issue).

Finally, the concluding implication of the present findings is related to the fact that the investigations of the response modality effect in the Stroop task seemingly contribute to uncovering the different components of Stroop interference. Even though these different components still remain to be further studied, namely, with more time- and, perhaps, locus-sensitive indicators, it seems rather obvious that the historically favored single-stage response competition accounts should be abandoned in favor of multistage accounts of Stroop interference (Risko et al., 2006; Augustinova et al., 2018b).

This paradigmatic shift is, indeed, important because single-stage response competition accounts still largely dominate both empirical research and clinical practice, such that many researchers and practitioners who are interested in Stroop interference itself and/or in its measurement still seem to be unaware that it goes far beyond a mere response competition and that it should, thus, be measured or at least interpreted as a composite phenomenon involving additional types of components. Consequently, the extended semantic Stroop paradigm used in the present study might turn out as an evaluation tool that is simple enough to be administered in both laboratory and field (i.e., clinical) settings. Indeed, the specific contribution of all three types of conflict (task, semantic, and response conflicts), as well as the modulation (or the lack of thereof) of these distinct contributions, can be clearly seen within this paradigm – at least when administered with vocal responses. Also, and importantly, it is not restricted to manual responses as is the case with the so-called 2-to-1 paradigm (De Houwer, 2003; see also, e.g., van Veen and Carter, 2005; Hasshim and Parris, 2014, 2015). As already emphasized by Augustinova et al. (2018b), the extended form of the Stroop paradigm can therefore “be administered not only using an item-by-item (i.e., computerized) presentation but also, potentially, in a card version that is still in widespread use in clinical practice (see, e.g., Bugg et al., 2007 for an example and Augustinova and Ferrand, 2014b; Augustinova et al., 2016 for discussions of this issue)” (p. 61, see also Augustinova et al., 2018b). Thus, a more fine-grained measurement of Stroop interference would represent an added value, namely, for neuropsychological practice. Indeed, because different components of Stroop interference are likely to be associated with distinct neural substrates (Bench et al., 1993; Milham et al., 2001; van Veen and Carter, 2005; Chen et al., 2013), it remains highly plausible that the different conflicts involved in Stroop interference are selectively impacted by various clinical conditions (e.g., Alzheimer’s disease, attention deficit hyperactivity disorder). The present study therefore motivates a comparison of the neural substrates of all three conflict types in the same neuroimaging study. Indeed, one of the rationales for the original proposal of a TC-RC account of Stroop interference by MacLeod and MacDonald (2000) lies in the observation that the Anterior Cingulate Cortext (ACC) appeared to be more activated by incongruent and congruent stimuli when compared to repeated letter neutral stimuli (e.g., XXX; see also Bench et al., 1993). That said, no study has yet directly investigated this possibility using the required contrast of color-neutral words to color-neutral letter strings, so the precise location of activation within the ACC associated with task conflict is not known (see Milham et al., 2001; van Veen and Carter, 2005; Chen et al., 2013 for distinct locations of semantic vs. response conflicts). Therefore, and again, future studies need to address this remaining issue directly. Meanwhile, the present study largely reaffirms that Stroop interference and facilitation have several loci as opposed to just a single (i.e., response) locus, at least with vocal responses.

## Data Availability

The datasets generated for this study are available on request to the corresponding authors.

## Ethics Statement

Human subject research: The studies involving human participants were reviewed and approved by Comité de Protection des Personnes Sud- Est 6 (Clermont- Ferrand, France). The patients/ participants provided their written informed consent to participate in this study. Animal subjects: No animal studies are presented in this manuscript. Human images: No potentially identifiable human images or data is presented in this study.

## Author Contributions

MA and LF designed the study, collected and analyzed the data, and wrote the first draft of the manuscript. All authors contributed to the interpretation of the data, critically revised the final version of the manuscript, and equally contributed to read, comment, and approve the submitted version.

## Conflict of Interest Statement

The authors declare that the research was conducted in the absence of any commercial or financial relationships that could be construed as a potential conflict of interest.

## Footnotes

1 As suggested by one of the reviewers, it is worth mentioning that the semantic conflict observed in the manual response condition was quite small. Specifically, its magnitude was 14 ms in the condition of high proportion and 8 ms in the condition of low proportion of neutral distractors (i.e., # signs) that was also manipulated in this study, although it did not significantly affect the aforementioned amplitudes of semantic conflict.

2 One participant made more than 33% of errors; the microphone did not detect responses for 2 participants, and EPrime failed to record responses for 1 participant.

3 The results of the same analysis on percentages of errors somewhat mirrored those observed on RTs as it revealed the significant main effect of stimulus type [*F*(3,210) = 25.70; *p* < 0.001, $\eta_{p}^{2}$ = 0.269] that was also included in the significant stimulus × response modality interaction [*F*(3,210) = 15.36; *p* < 0.001, $\eta_{p}^{2}$ = 0.180] with the main effect of response modality remaining non-significant [*F*(1,70) = 1.08; *p* = 0.302, $\eta_{p}^{2}$ = 0.015]. As can be seen in Table 1, the decomposition of the overall interaction suggests that all types of items were equally error prone in manual response modality (all *p*_s_ ≥ 0.418), whereas standard color-incongruent items were significantly more error prone than the other kinds of items (all *p*_s_ < 0.001) in vocal response modality. In sum, these results are not only in line with past studies but also rule out the possibility of speed–accuracy trade-off.

4 This exclusion was due to the fact that in the vocal response modality, eight participants exhibited more than 33% of errors and/or no responses (because the microphone did not detect their responses), and 1 participant made irrelevant mouth/tongue movements that systematically triggered the voice key prematurely.

5 The results of the same analysis on percentages of errors somewhat mirrored those observed on RTs as it revealed a significant main effect of Stimulus-type [*F*(5,175) = 32.12; *p* < 0.001, $\eta_{p}^{2}$ = 0.479] that was also included in the significant Stimulus-type × Response-modality interaction [*F*(5,175) = 14.97; *p* < 0.001, $\eta_{p}^{2}$ = 0.300] with the main effect of Response-modality remaining non-significant [*F*(1,35) = 2.07; *p* = 0.158, $\eta_{p}^{2}$ = 0.056]. As can be seen in Table 1, the decomposition of the overall interaction suggests that standard color-incongruent items were significantly more error-prone than the other types of items (all *p*_s_ < 0.001) in the vocal modality; it was also the case in the manual response modality (all *p_s_* < 0.03), except for the difference between standard color-incongruent items and associated color-incongruent items (*p* = 0.34).
